# Intelligence, Correspondence, &c.

**Published:** 1832-04-01

**Authors:** 


### INTELLIGENCE, CORRESPONDENCE, &c.

The Index and Title-page of this volume has been unavoidably deferred till next Number.

*** In consequence of an *addition* of 72 pages, of the smallest type, to this Number, we are compelled to tax our subscribers with one shilling extra; a tax which does not defray the paper and letter-press, leaving out of the question the expense of literary composition, which has been peculiarly heavy this quarter. We venture to say, that none of our subscribers will complain of the price of this Number of the Journal.

*Literary Announcement.*

Mr. SCHLOSS informs us that he intends to publish a new Work, entitled the Anatomy of the Horse, by Professor GURLT, of Berlin. It is to be translated from the German. For remarks on Mr. Schloss's Plates of Human Anatomy, we refer to the Bibliographical Record.

The name of the Surgeon at Banbury, who details the case of cholera there, is Mr. BUSBY: the communicant Mr. H., not Mr. J. CLARKE, of Lamb's Conduit-street.

